# Granulomatous Prostatitis Associated with Intravesical Bacillus Calmette Guerin Therapy

**DOI:** 10.1590/0037-8682-0561-2023

**Published:** 2024-02-05

**Authors:** Elif Gündoğdu

**Affiliations:** 1 Eskişehir Osmangazi University, Faculty of Medicine, Department of Radiology, Eskişehir, Turkey.

A 65-year-old male patient with a history of urothelial carcinoma of the bladder was referred for multi-parametric prostate magnetic resonance imaging (MRI) due to an indurated prostate gland on digital rectal examination and elevated prostate-specific antigen (PSA) level during follow-up. His medical history was otherwise unremarkable except for the urothelial carcinoma of the bladder. He had previously received intravesical Bacillus Calmette-Guérin (BCG) therapy for his condition. While the PSA level was within the normal range (3.19 ng/mL) ​​one year prior, it had now increased to 9.1 ng/mL. 

**FIGURE 1:**
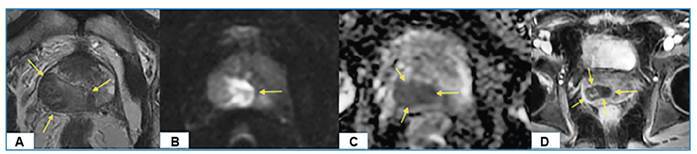
Multi-parametric prostate magnetic resonance imaging (MRI) findings. (A) T2-weighted image shows a heterogeneously hypointense lesion (yellow arrows) in the lateral-posterolateral section of the right peripheral gland. (B) Diffusion-weighted image demonstrates diffusion restriction within the lesion (yellow arrow). (C) The apparent diffusion coefficient (ADC) map reveals low ADC values corresponding to the lesion. (D) The post-contrast T1-weighted image depicts peripheral ring enhancement (yellow arrows) within the lesion.

Multi-parametric prostate MRI revealed a heterogeneous hypointense lesion on T2-weighted images in the lateral-posterolateral section of the right peripheral gland. The lesion demonstrated diffusion restriction and low apparent diffusion coefficient values on diffusion MRI. Following contrast agent administration, peripheral ring enhancement was observed within the lesion (Figure 1). 

Considering the patient’s history of intravesical BCG treatment, granulomatous prostatitis and prostate cancer were included in the differential diagnosis. Anti-tuberculosis (TB) therapy was initiated, resulting in a subsequent decrease in PSA levels. Histopathology following radical cystoprostatectomy performed two years later for recurrent urothelial carcinoma confirmed the presence of a granuloma in the prostate gland. 

Granulomatous prostatitis is a rare, chronic inflammatory disease of the prostate^1^. The prevalence of granulomatous prostatitis after BCG therapy has been reported to range between 1.3-40%^2^. It can mimic prostate cancer both clinically and radiologically. A history of intravesical BCG treatment, peripheral ring enhancement on MRI, and response to anti-TB therapy can help distinguish between the two conditions.
